# Squeezing the Muscle: Compression Clothing and Muscle Metabolism during Recovery from High Intensity Exercise

**DOI:** 10.1371/journal.pone.0060923

**Published:** 2013-04-17

**Authors:** Billy Sperlich, Dennis-Peter Born, Kimmo Kaskinoro, Kari K. Kalliokoski, Marko S. Laaksonen

**Affiliations:** 1 Department of Sport Science, University of Wuppertal, Wuppertal, Germany; 2 Department of Sport Science, University AF Munich, Munich, Germany; 3 Turku PET Centre, University of Turku, Turku, Finland; 4 Anesthesiology and Intensive Care, University of Turku, Turku, Finland; 5 Swedish Winter Sports Research Centre, Department of Health Sciences, Mid Sweden University, Östersund, Sweden; Wageningen University, The Netherlands

## Abstract

The purpose of this experiment was to investigate skeletal muscle blood flow and glucose uptake in m. biceps (BF) and m. quadriceps femoris (QF) 1) during recovery from high intensity cycle exercise, and 2) while wearing a compression short applying ∼37 mmHg to the thigh muscles. Blood flow and glucose uptake were measured in the compressed and non-compressed leg of 6 healthy men by using positron emission tomography. At baseline blood flow in QF (P = 0.79) and BF (P = 0.90) did not differ between the compressed and the non-compressed leg. During recovery muscle blood flow was higher compared to baseline in both compressed (P<0.01) and non-compressed QF (P<0.001) but not in compressed (P = 0.41) and non-compressed BF (P = 0.05; effect size = 2.74). During recovery blood flow was lower in compressed QF (P<0.01) but not in BF (P = 0.26) compared to the non-compressed muscles. During baseline and recovery no differences in blood flow were detected between the superficial and deep parts of QF in both, compressed (baseline *P* = 0.79; recovery *P* = 0.68) and non-compressed leg (baseline *P* = 0.64; recovery *P* = 0.06). During recovery glucose uptake was higher in QF compared to BF in both conditions (P<0.01) with no difference between the compressed and non-compressed thigh. Glucose uptake was higher in the deep compared to the superficial parts of QF (compression leg P = 0.02). These results demonstrate that wearing compression shorts with ∼37 mmHg of external pressure reduces blood flow both in the deep and superficial regions of muscle tissue during recovery from high intensity exercise but does not affect glucose uptake in BF and QF.

## Introduction

Skeletal muscle blood flow incorporates a key role in aerobic muscle metabolism matching the delivery of oxygen and energy substrates for energetic demands, as well as the transportation of waste products and heat from the muscle tissue. The response of skeletal muscle blood flow and metabolism at the onset of and during exercise are well documented. In general, muscle blood flow increases rapidly with an exercise-depended plateau after approximately 30 s of exercise [1], [2]. Muscle blood flow is heterogeneously distributed [3] in a manner that it is higher in the deeper compared to the superficial parts of the m. quadriceps femoris [4].

Another point is that blood glucose concentration plays an important role in restoring muscle glycogen during recovery from exercise [5]. Unfortunately, little is known about the crucial role of blood flow and its association to glucose uptake, during recovery from high intensity exercise [6] and so far no study investigated this matter in connection with the application of compression clothing.

In the past two decades, various forms of compression clothing have been applied by elite and recreational athletes due to accumulating evidence regarding the possible performance [7], [8] and recovery [9]–[11] enhancing properties. Early research showed that the application of 15 mmHg external pressure while lying in supine position reduces the cross-sectional area of the venous systems (from 2.65 cm^2^ to 0.53 cm^2^) thereby elevating mean linear blood flow velocity (from 0.5 cm/s to 2.5 cm/s) [12] and thus reducing contact time and thrombogenesis [13]. The application of compression seems to enhance muscle blood flow [14] and has been associated with enhanced clearance of metabolites, such as blood lactate [15], [16]. However, it also has been suggested that reduced levels of blood lactate are attributable to a greater retention of the molecule within the muscle [15], [16]. In this context two recent literature reviews summarized the effects of compression on metabolite clearance to be controversial [17], [18]. In this regard, especially clothing with a high level of compression (>30 mmHg) has not been investigated regarding its effects on blood flow and glucose uptake during recovery.

Positron emission tomography (PET) provides a unique tool to investigate metabolism in the entire muscle tissue noninvasively *in vivo* [19]. The PET method is based on the use of short-lived positron emitting radioisotopes and thus, allows quantitative measurements of tracer concentrations in the muscle tissue providing important information on muscle metabolism. Therefore, the PET method will reveal regional differences within the muscle when wearing compression clothing.

Therefore the primary aims of this experiment were to 1) study skeletal muscle blood flow and glucose uptake during recovery from high intensity exercise, and 2) investigate whether compression clothing enhances skeletal muscle blood flow and glucose uptake.

## Methods

### Subjects and Ethics Statement

Six healthy males volunteered for this study. Their age, anthropometric characteristics and peak oxygen uptake values are summarized in Table 1. All participants were informed about the purpose, nature and potential risks of the study, and gave their written informed consent prior the study. All subjects were instructed to be adequately hydrated and to refrain from consuming alcohol, caffeine and exercise for 24 h, and food for 3 h before the test. All procedures were approved by the Ethical Committee of the Hospital District of South-Western Finland and conducted in accordance with the Declaration of Helsinki.

**Table 1:** Study subject characteristics.

ID	Body mass[kg]	Stature [cm]	Age [years]	VO_2peak_ [ml/min/kg]
1	80	191	22	55
2	68	180	20	64
3	72	174	24	47
4	72	180	21	52
5	74	190	21	56
6	68	176	26	51
Mean±SD	72±4	181±7	22±2	54±6

### Study Design

**Figure 1:**
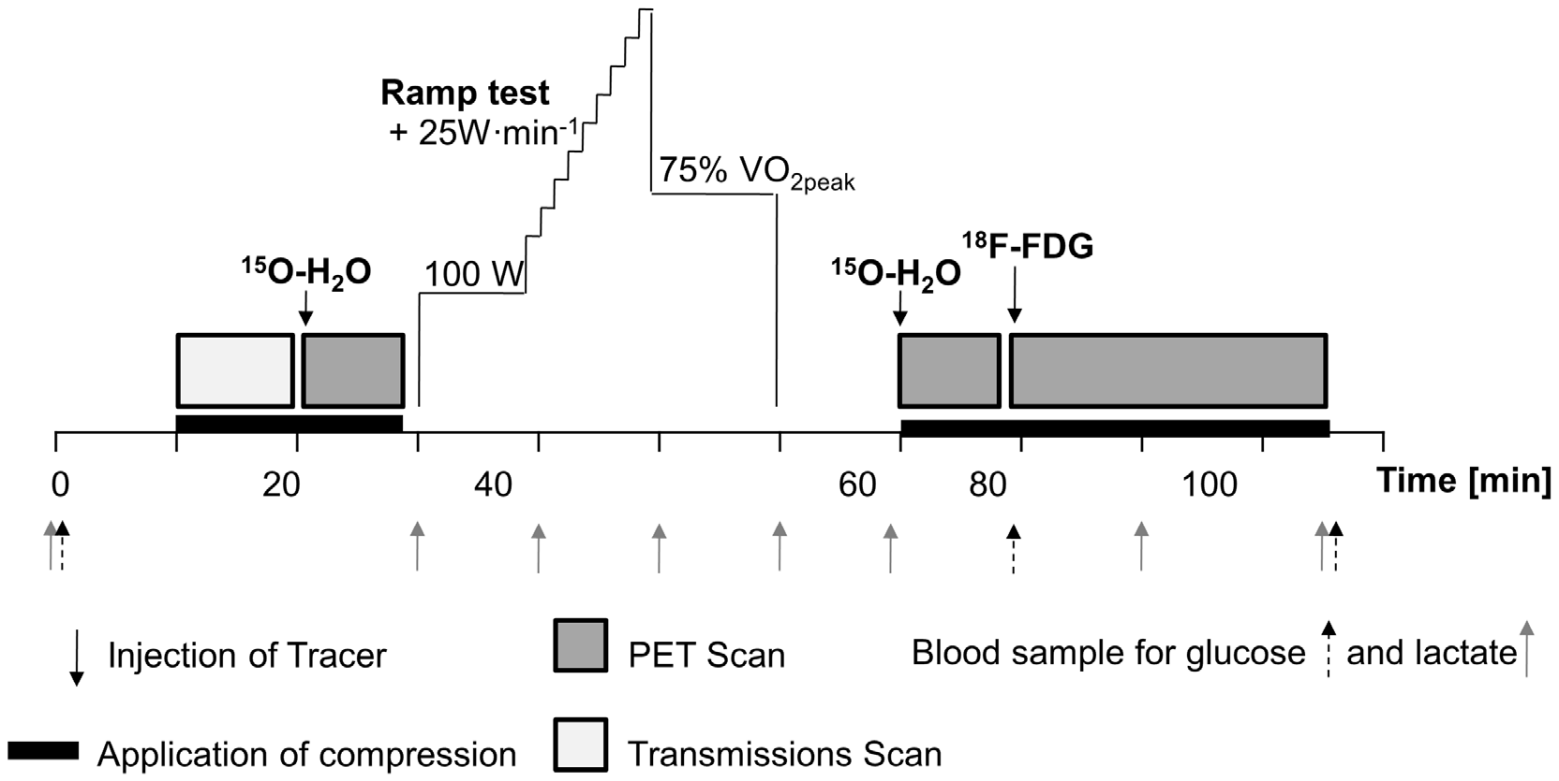
Study design. A schematic illustration with all time points of measurements.

The present study consisted of baseline measurements, 10-min warm-up, high intensity cycling to exhaustion, 1-min of recovery followed by cycling at 75% of peak oxygen uptake and post-exercise measurements. Figure 1 illustrates the study design with all time points of measurements.

The baseline measurements included the assessment of anthropometric characteristics, blood sampling in supine position with basic hemogram as final check of the subjects’ hematological health, the fitting and measurement of the compression-shorts, and PET-scanning for muscle blood flow. The high intensity exercise protocol consisted of 10 min cycling at 100 W (warm-up) which was immediately followed by a ramp test to exhaustion (+25 W/min) in order to measure the peak power output and peak oxygen uptake. After 1 min of passive recovery the subjects continued to cycle at 75% of peak oxygen uptake. The 10-min warm-up, followed by the high intensity exercise protocol together with the passive 1-min recovery and cycling at 75% of peak oxygen uptake altogether lasted 30 min Venous blood was sampled after the warm-up, 1 min after the termination of the ramp test, and at the end of the 30-min exercise protocol (at 30 min). Post-exercise measurements included blood sampling as well as PET-scanning for muscle blood flow and glucose uptake.

### Measurement of Muscle Blood Flow and Glucose Uptake

Before any measurements two catheters were inserted, one into an antecubital vein for saline infusion and injection of tracer, and another into the opposite radial artery for blood sampling. Thereafter, the subjects were fastened to the PET scanner [20] and the scanning area (thigh) was carefully marked on the skin in order to secure the same scanning position each time. After the transmission scan for photon attenuation correction an intravenous injection of ^15^O-H_2_O (772±55 MBq) was given immediately followed by a dynamic PET scanning (6 min) for measuring muscle blood flow. Immediately after the exercise protocol, the subject was positioned to the same position in the PET scanner and muscle blood flow was measured 10 min after the cessation of exercise in similar manner as explained above. Muscle glucose uptake was measured only during recovery using ^18^F-FDG (2-[(18)F]fluoro-2-deoxy-D-glucose) tracer (185±13 MBq) which was given 20 min after the cessation of exercise and followed by a PET scan for 25 min. The positron-emitting tracers, ^15^O-H_2_O and ^18^F-FDG, were produced as previously described [21], [22].

An ECAT EXACT HR+ scanner (Siemens/CTI, Knoxville, TN) was used for emission scanning. The scanning area was over both thighs thus including most part of thigh muscles. All PET data were collected and processed as previously described [20], [23]–[25]. Blood flow was calculated using an autoradiographic method as mentioned earlier [20], [25]. Muscle glucose uptake was analysed using the fitting method by Patlak [26].

### Regions of Interest

Regions of interest (ROIs) surrounding the extensors of the femoral muscles (m. quadriceps femoris, QF) and posterial part of the thigh muscles (m. biceps femoris, BF) were drawn into 20 subsequent cross-sectional planes in both thighs as previously described [3]. Additionally two ROIs were drawn in QF muscle and are defined as superficial (the superficial half of QF) and as deep (the deeper half of QF).

### Other Measurements and Calculation

During the high intensity exercise protocol all subjects were equipped with an portable breath-by-breath analyzer (Cortex Metamax 3B, Leipzig, Germany) using standard algorithms with dynamic account for the time delay between the gas consumption and volume signal. The system was calibrated prior to each test using calibration gas (15.8% O_2_, 5% CO_2_ in N; Praxair, Germany), targeting the range of anticipated fractional gas concentration, and a precision 3l syringe (Cortex, Leipzig, Germany). The participants breathed through a Hans-Rudolph mask and a turbine flow meter during all testing. A heart rate belt (Polar S31, 1 Hz) was linked to the portable breath-by-breath system and time aligned with the respiratory data.

### Compression Clothing

During baseline and recovery from high intensity exercise all subjects wore compression shorts extending from above the hip to above the knee (Sigvaris, Winterhur, Switzerland; 94% Polyamide and 6% Lycra), while the cycling exercise was performed without any compression. The garments were custom-made and fitted to previously measured thigh girth. At baseline one of the trouser-legs (left or right) was cut distally from the participants’ knee up to femoral bone neck in order to induce zero pressure on the thigh (non-compression). On the other leg (compression) the mean pressure on four sites of the thigh at its maximum girth was aimed to be 35 mmHg. The level of compression applied to each individual was pre-checked before each test five times, according to international recommendations [27]. For this a pneumatic sensor (SIGaT®, Ganzoni, St. Gallen, Switzerland) was used to receive the in-vivo pressure dimensions as described previously [28]. Compression was applied to one of each subjects’ legs. The application of compression and non-compression to either the left or right leg was randomly chosen. The cycling exercise was performed without any compression.

### Statistical Analyses

All data were calculated with conventional procedures and presented as mean values and standard deviation (SD). All data were checked for normality as well, with no necessity for further transformation. ANOVA for repeated measurements was performed to test the significance of differences between the clothing conditions (i.e., with or without compression) and the point of measurement (i.e. baseline or recovery). After detection of global significance a Fisher *post hoc* procedure was employed. An alpha level of *P*<0.05 was considered to be statistically significant. Effect sizes from the various outcome measures were calculated using Cohen’s d for the detection of the practical application and meaningfulness of the findings. According to standard practice thresholds, for small, medium and large were defined as 0.20, 0.50 and 0.80, respectively [29]. All analyses were carried out with the Statistica software package for Windows® (version 7.1, StatSoft Inc., Tulsa, OK, U.S.A).

## Results

The mean pressure at the compression over the thigh muscle was 36.7±4.1 mmHg. The regional pressure values are illustrated in Table 2.

**Table 2:** All subjects’ pressure of the shorts at different areas of the thigh.

ID	Pressure [mmHg]			
	M. rectus femoris	M. vastus lateralis	M. vastus medialis	M. biceps Femoris
1	36	40	34	41
2	35	41	43	42
3	39	41	44	38
4	36	33	31	37
5	31	33	29	38
6	34	36	32	36
Mean±SD	35±3	37±4	36±6	39±2

The mean peak power output for all subjects during the ramp test was 350±22 W with a corresponding peak oxygen uptake of 3913±434 ml/min (54.1±5.7 ml/kg/min). This high intensity effort resulted in peak levels of plasma lactate of 11.2±2.0 mmol/l.

**Figure 2:**
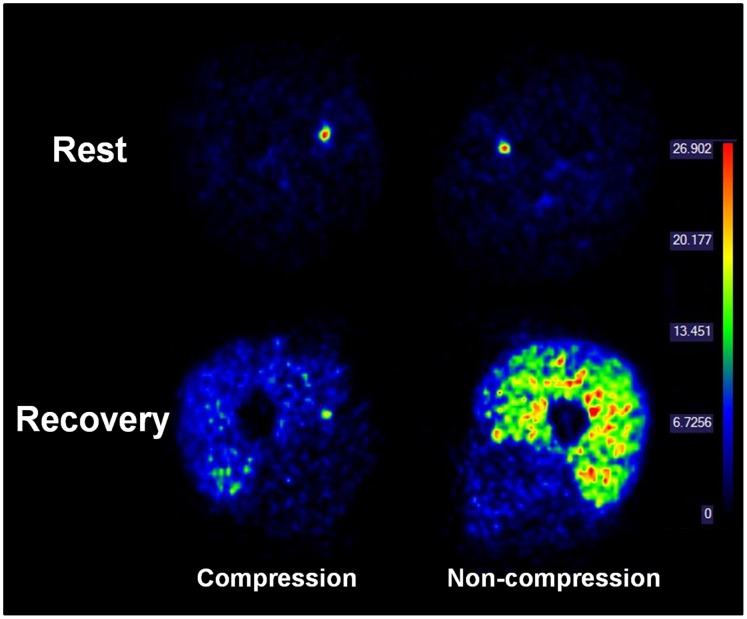
A representative example of muscle blood flow PET image before (rest) and after (recovery) the high intensity exercise. This figure illustrates that blood flow is lower in compressed compared to non-compressed leg. The color bar on the right side of the figures represents the quantitative scale for blood flow values.

**Figure 3:**
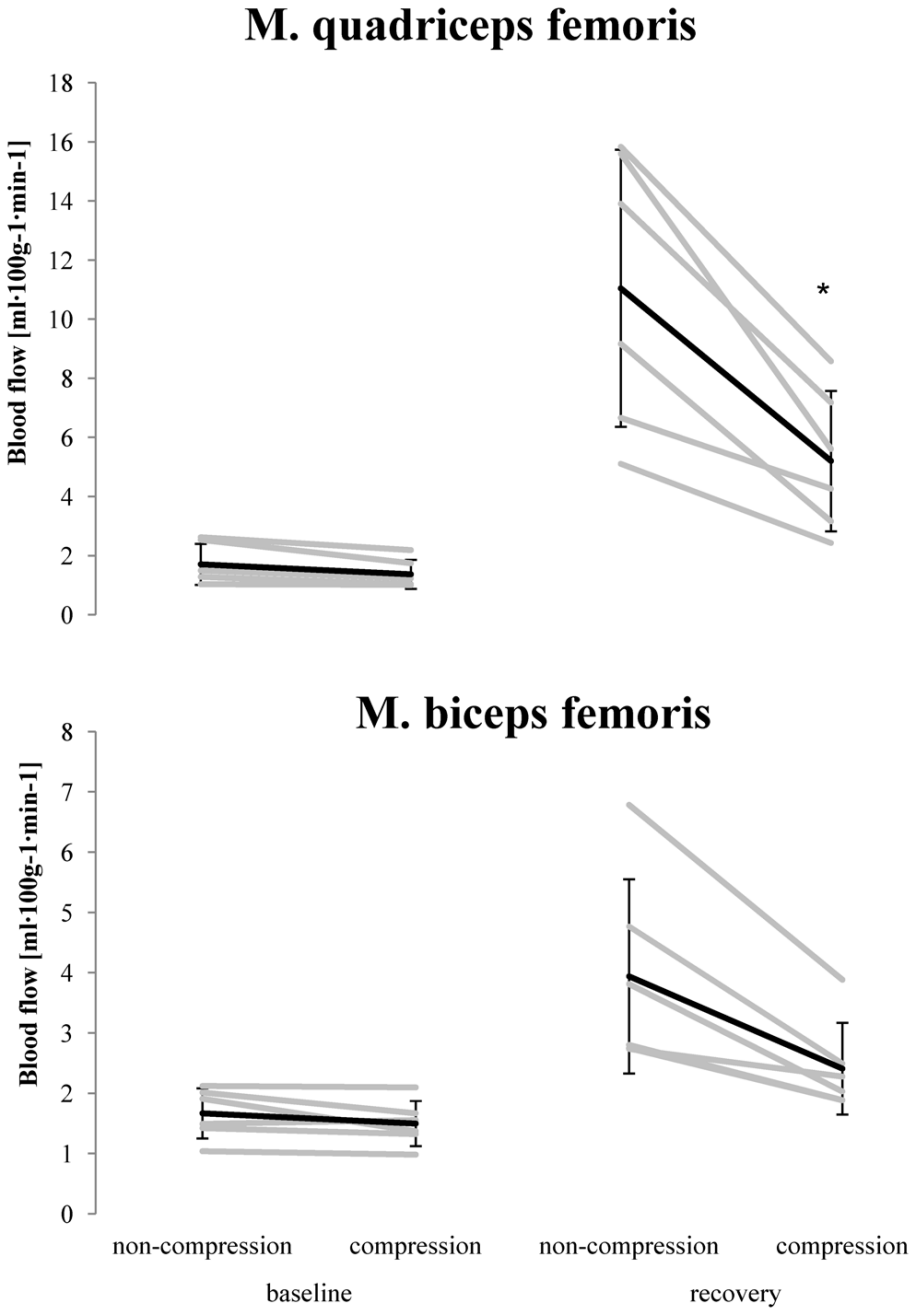
Skeletal muscle blood flow. M. quadriceps femoris (above) and m. biceps femoris (below) without (non-compression) and with compression (compression) during baseline and recovery from high intensity exercise (*indicates statistical significant differences *P*<0.05).

A representative example of muscle blood flow PET image before (rest) and after (recovery) the high intensity exercise is illustrated in Figure 2. At baseline blood flow in QF was unchanged with compression compared to the non-compressed leg (*P* = 0.79; effect size = 0.49) (Figure 3). No differences were detected between compression and non-compression in BF (*P* = 0.90; effect size = 0.50). During recovery muscle blood flow was higher compared to baseline in both compressed (*P* = 0.002; effect size = 3.16) and non-compressed QF (*P*<0.001; effect size = 3.94) but not in compressed (*P* = 0.41; effect size = 2.15) and non-compressed BF (*P* = 0.05; effect size = 2.74). In addition, during recovery blood flow was lower in compressed QF (*P*<0.001; effect size = 1.55) but not in BF (*P* = 0.26; effect size = 1.19) when compared to same muscles in the non-compressed leg (Figure 3). During baseline and recovery no differences in blood flow were detected between the superficial and deep parts of QF in both, compressed (baseline *P* = 0.79; recovery *P* = 0.68) and non-compressed leg (baseline *P* = 0.64; recovery *P* = 0.06).

**Figure 4:**
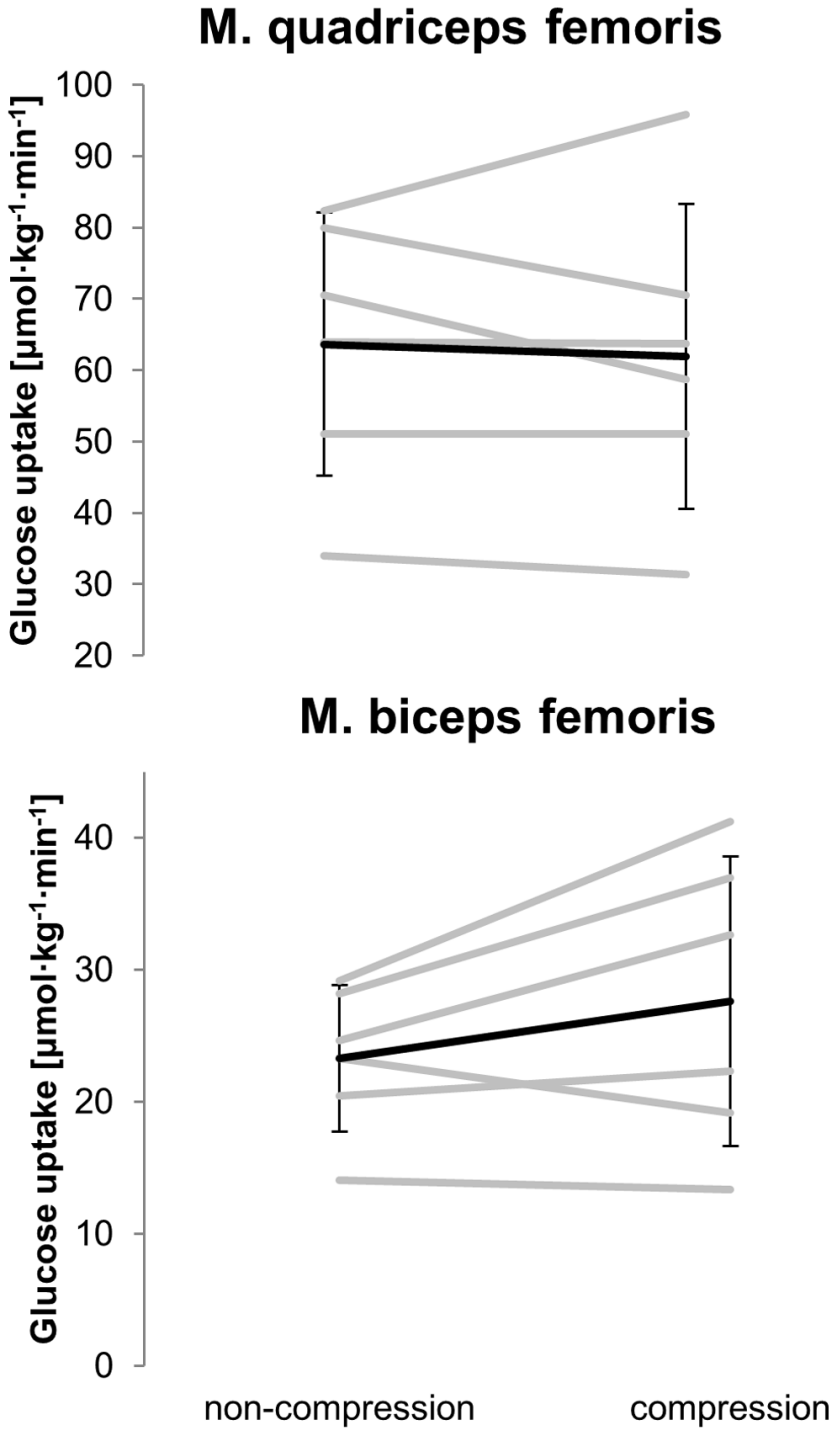
Glucose uptake. M. quadriceps femoris (above) and m. biceps femoris (below) without and with compression during recovery from high intensity exercise.

During recovery muscle glucose uptake was higher in QF compared to BF in both conditions (compressed leg *P*<0.01, effect size = 2.85 and non-compressed leg *P*<0.01, effect size = 4.19) (Figure 4). However, in both QF (*P* = 0.88, effect size = 0.09) and BF (*P* = 0.70, effect size = 0.49) no difference was observed in glucose uptake between the compressed and non-compressed thigh. Glucose uptake was higher in the deep compared to the superficial parts of QF in both conditions (Table 3) (compression leg *P* = 0.02, effect size = 0.33; non-compression leg *P* = 0.02, effect size = 0.78).

**Table 3:** Skeletal muscle blood flow and glucose uptake in superficial and deep parts of m. quadriceps femoris (n.d. = not determined).

	Baseline				Recovery			
	Compression	Non-compression	*P*	*d*	Compression	Non-compression	*P*	*d*
Blood flow [mL/100 g/min]								
Superficial	1.2±0.4	1.4±0.6	0.92	0.55	5.1±2.4	9.8±4.2	0.05	1.94
Deep	1.5±0.6	2.0±0.9	0.82	0.92	5.7±2.8	12.4±5.2	0.01	2.27
Glucose uptake [µmol/kg/min]								
Superficial	n.d.	n.d.			55.3±15.8	56.8±15.6	0.96	0.14
Deep	n.d.	n.d.			70.4±27.5	71.2±22.9	0.93	0.04

## Discussion

The present results show that skeletal muscle blood flow is higher during the acute phase of recovery from high intensity exercise (∼15 min post exercise) when compared to baseline levels in m. quadriceps femoris but not in m. biceps femoris. When applying ∼37 mmHg external compression to the thigh muscle, blood flow during recovery is decreased but glucose uptake seems to be unchanged.

Earlier investigations have shown that compression garments appear to enhance blood flow in the venous system [30], [31] and increase arterial inflow [32], resulting in improved peripheral circulation. Therefore it was hypothesized that compression applied to the thigh muscle after high intensity exercise might lead to increased blood flow thus enhancing the glucose uptake due to increased glucose delivery. However, according to the present results shorts with ∼37 mmHg of external pressure decreased muscle blood flow during recovery from high intensity exercise and did not seem to affect the glucose uptake in the same muscles. It is noteworthy that the previous study by Bochmann et al. [32] measured the arterial inflow with venous occlusion plethysmography at the forearm muscles and applying 13 to 23 mmHg of compression. Therefore the conflicting data when compared to our results may result from the different test procedures, the lower level of compression and the different muscles involved.

While lying horizontal the application of 15 mmHg on an inactive muscle diminishes the cross-sectional area of the venous systems by 80% (from 2.65 cm^2^ to 0.53 cm^2^) and improves mean linear blood flow velocity 5-fold [12]. The reduction in the cross sectional area due to external pressure is proposed to compress superficial limb tissues, thereby compressing underlying veins, and by this reduce pooling [17]. Accordingly, the applied compression is assumed to reduce the transmural pressure in local arterioles [33] thereby leading to vasodilatation by myogenic regulation [34] and increases in blood flow in underlying tissue [32]. Although it is obvious that the cross sectional area of the thigh was reduced during compression (not determined, but please see Figure 2), our data obtained during the acute phase of recovery from high intensity exercise do not support the hypothesis that the compression shorts of ∼37 mmHg improves blood flow during recovery from high-intensity exercise.

In exercise science, there exists no common consent on the level and type of compression to enhance performance and recovery [17], [18]. In the medical field however, several classes of compression hosiery are recommended ranging from moderate (Class I: 18–21 mmHg), medium (Class II: 23–32 mmHg), strong (Class III: 34–46 mmHg) to very strong (Class IV: >49 mmHg) [35]. In the present study we applied a pressure of ∼37 mmHg to the muscle’s belly which would be equivalent to a class III pressure. Based on the findings of a recent review the mean pressure in recovery related exercise studies ranged from 10–30 mmHg [11], [36]–[38]. Thus, the pressure applied in the present study is higher compared to other recovery studies. Based on the present data the external compression of the thigh muscle at this pressure seems to lead to a mechanical hindrance in muscle blood flow. This is supported by earlier investigations which have shown that during static muscle contractions (>10% of maximal voluntary contraction force) blood flow is impaired and thereby affects muscle oxygen delivery [39]. In addition, we observed that muscle blood flow was also decreased in the deeper parts of QF muscle. Thus, compressions garments applying an external pressure level of ∼37 mmHg affects the muscle blood flow not only in superficial but also in the deeper parts of the muscle tissue.

Previous research describes several mechanisms on the function of compression textiles for recovery purposes. Muscle swelling [40], [41] may arise after high-intensity exercise due to structural damage to the muscles’ contractile elements accompanied with inflammatory response [40] and elevated tissue osmotic pressure [42] finally resulting in edema [42]. The application of compression is thus supposed to reduce exercise-induced edema by stimulating lymphatic outflow and transporting fluid from the muscles’ interstitium back into the circulation [42], [43]. However the present findings do not support this, since decreased blood flow in the muscle tissue was observed in the compressed QF muscle during recovery from high intensity exercise. One potential explanation for the mixed findings is that the exposure to compression may not have been long enough, since some studies evidence changes in muscle damage markers [9], [10], [38], [42], [44] and further performance indicators such as strength and power measures 24 and 48 h after high intensity exercise [10], [11], [37].

One of the most novel aspects of the present study is that, under the applied conditions, muscle glucose uptake remains unchanged despite reduced blood flow during the recovery from high intensity exercise. Glucose uptake is a complex process and depends mainly on the GLUT-4 glucose transporter concentration [45] and the glucose phosphorylation level in the muscle cell [46] but also on the glucose delivery to the working muscles [1], [46]. In this context it has been shown that plasma glucose concentration is highest ∼40 min after exercise at 65% peak O_2_ consumption [47] and that glucose uptake in QF is higher at 75% VO_2peak_ compared to cycling at 30% and 55% of VO_2peak_ [48]. During exercise hyperemia increases muscle capillary blood flow significantly [46], [49], and thereby leads to a higher glucose delivery to the working muscle. This hemodynamic effect favors the uptake of glucose. However, the present data indicates that glucose delivery was decreased during recovery from high intensity exercise when wearing compression shorts due to reduced muscle blood flow. Accordingly, while the absolute amount of glucose extracted remains unchanged, the extraction of glucose from blood must be increased relative to its delivery. Indeed, it has been shown that the level of glucose extraction during low intensity exercise is rather low (∼15–20%) [50] specifying a relatively large reserve to increase glucose uptake without any increase in delivery. Therefore, unchanged glucose uptake due to compression indicates that the glucose uptake is not affected by the use of compression clothing during recovery from high intensity exercise. However, glucose uptake was higher in the deeper parts of the QF muscle. This finding is well in line with other findings [51] showing during dynamic knee-extension exercise that glucose uptake was higher in m. vastus intermedius and m. vastus medialis compared to m. vastus lateralis and m. rectus femoris.

From a methodological point of view the number of subjects in the present study was relative small but in the range of studies in this area [48], [52]. Therefore, more subjects would have given more statistical power for the data interpretation. However, the results especially for blood flow (Figure 3) and glucose uptake (Figure 4) were nearly identical in all subjects. In this case, from an ethical point of view, we refrained from recruiting additional subjects. Finally, in addition to the statistical analysis, we performed effect size calculation which supported in interpreting our data.

In conclusion, there are two novel findings in the present study. First, this study demonstrates that wearing compression shorts with ∼37 mmHg of external pressure reduces blood flow both in the deep as well as superficial regions of muscle tissue during the acute recovery phase from high intensity exercise. Second, muscle glucose uptake was unchanged and thus independent from blood flow. We therefore conclude that wearing compression shorts with ∼37 mmHg during recovery from high intensity exercise does not elevate muscle blood flow and therefore does not lead to a greater delivery of energy substrates or enhanced muscle glucose uptake when compared to non-compression clothing.
